# Intense ultraviolet emission from needle-like WO_3 _nanostructures synthesized by noncatalytic thermal evaporation

**DOI:** 10.1186/1556-276X-6-451

**Published:** 2011-07-13

**Authors:** Sunghoon Park, Hyunsu Kim, Changhyun Jin, Chongmu Lee

**Affiliations:** 1 Department of Materials Science and Engineering, Inha University, 253 Yonghyeon-dong, Nam-gu, Incheon 402-751, Republic of Korea

## Abstract

Photoluminescence measurements showed that needle-like tungsten oxide nanostructures synthesized at 590°C to 750°C by the thermal evaporation of WO_3 _nanopowders without the use of a catalyst had an intense near-ultraviolet (NUV) emission band that was different from that of the tungsten oxide nanostructures obtained in other temperature ranges. The intense NUV emission might be due to the localized states associated with oxygen vacancies and surface states.

## Background

Tungsten oxide is of particular interest owing to its outstanding electrochromic, optochromic, and gas chromic properties [1-3], which make it a promising candidate for applications in smart windows, wide-angle high-contrast displays, gas, and temperature sensors [4-6]. Tungsten oxide in bulk form has been studied extensively over the past few decades. Nevertheless, there are relatively few reports on tungsten oxide nanostructures. In particular, little is known about the luminescence properties of tungsten oxide nanostructures possibly because tungsten oxide is an indirect band gap semiconductor with low-emission efficiency. Two strong emissions from tungsten oxide nanostructures, near-ultraviolet (NUV) emission and blue emission, have been reported [7-12]. Nevertheless, there is still some controversy regarding the origins of the two emissions. Niederberger *et al*. [7] suggested that the blue emission from WO_3 _nanoparticles in an ethanol solution was due to a band-to-band transition. Luo *et al*. [8] also reported that the NUV and blue emissions from the WO_3 - *x *_nanowire network were due to the state of oxygen vacancies and a band-to-band transition, respectively. On the other hand, several reports have suggested the opposite. Lee *et al*. [9] and Feng *et al*. [10] reported that the NUV emission was attributed to a band-to-band transition; whereas, the blue emission was due to the localized states of oxygen vacancies or defects. Chang *et al*. [11] also suggested that the blue emission from nitrogen-doped tungsten oxide nanowires was due to oxygen vacancies.

In recent years, one-dimensional (1D) nanostructures have been investigated extensively owing to their interesting properties and potential applications in electronics and optoelectronics. A range of methods have been used to synthesize tungsten oxide 1D nanostructures, such as thermal oxidation, thermal evaporation, chemical vapor deposition, hydrothermal reaction, electrochemical techniques, aid of intercalated polyaniline, solution-based colloidal approach, and a combination of electrospinning and sol-gel techniques [13]. Of these, thermal evaporation might be the most attractive technique with the advantage of synthesizing a range of tungsten oxide nanostructures depending on the substrate temperature at lower temperatures than other techniques. This paper reports a simple novel thermal evaporation technique to obtain tungsten oxide nanostructures with a range of morphologies and sizes using a single apparatus and a single process and an intense ultraviolet emission from the needle-like tungsten oxide nanostructures grown in the temperature zone from 590 to 750°C by thermal evaporation.

## Experimental

**Figure 1:**
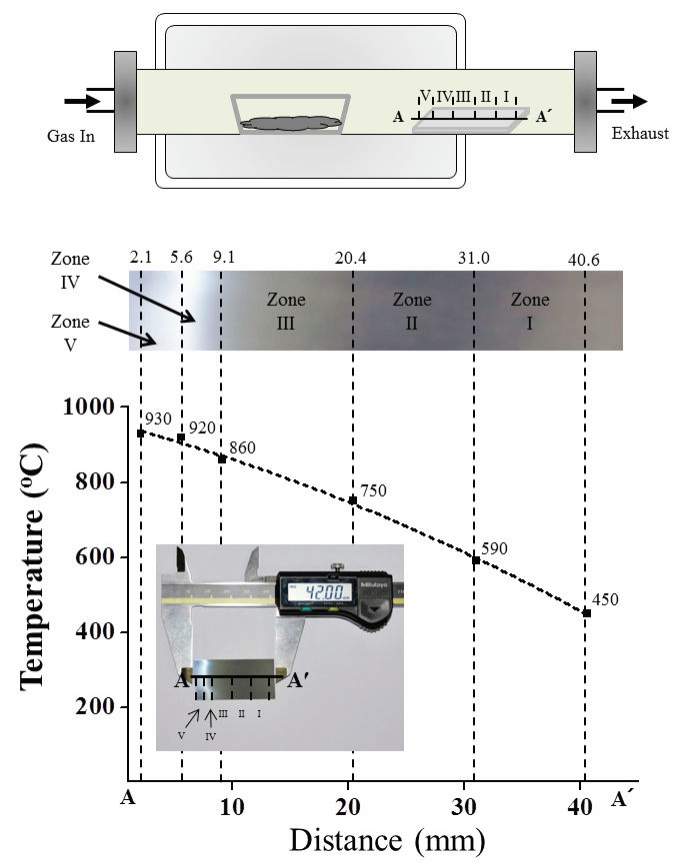
**Thermal evaporation process**. **(a) **Schematic diagram of the thermal evaporation system used to synthesize the tungsten oxide nanostructures. **(b) **Temperature versus substrate position showing five different substrate temperature zones.

Tungsten oxide nanostructures were synthesized by a thermal evaporation technique without a catalyst. The thermal evaporation process was carried out in a conventional horizontal tube furnace, as shown in Figure 1. An alumina boat with a length of 4 cm and a diameter of 1.5 cm containing a mixture of WO_3 _and graphite powders (1:1) were placed at the center of the quartz tube, and five pieces of P-type Si(100) wafer used as substrates were placed in five different temperature zones approximately 12 cm away from the alumina boat in the downstream direction: zone 1 (450°C to 590°C), zone 2 (590°C to 750°C), zone 3 (750°C to 860°C), zone 4 (860°C to 920°C), and zone 5 (920°C to 930°C). After arranging the substrates, the tube was pumped down to 10^-3 ^Torr using a rotary pump. High-purity nitrogen, and oxygen gases were introduced into the tube at flow rates of 200 and 5 sccm, respectively, throughout the entire synthesis process. The furnace temperature was increased to 1,050°C at a heating rate of 30°C/min. After being maintained at 1,050°C for 1 h, the furnace was cooled to room temperature, and the products were removed. During synthesis, the temperature in each of the five different zones was monitored using a thermocouple.

The collected nanostructure samples were characterized by scanning electron microscopy (SEM, Hitachi S-4200, Hitachi Ltd., Tokyo, Japan), transmission electron microscopy (TEM, Philips CM-200, Koninklijke Philips Electronics N.V., Amsterdam, Netherlands) equipped with an energy-dispersive X-ray spectrometer, and X-ray diffraction (XRD, Philips X'pert MRD diffractometer, Koninklijke Philips Electronics N.V., Amsterdam, Netherlands). The samples used for characterization were dispersed in absolute ethanol and ultrasonicated before the SEM and TEM observations. Glancing angle (0.5°) XRD was performed to examine the phases of the products obtained. Photoluminescence (PL) measurements were conducted at room temperature by using a SPEC-1403 PL spectrometer (HORIBA Ltd., Tokyo, Japan) with a He-Cd laser (325 nm) as the excitation source. The power of the He-Cd laser was 55 mW, and the diameter of the focal spot was 1 mm. Thus, the power density at the surface of the sample surface was approximately 7 W/cm^2^.

## Results and discussion

**Figure 2:**
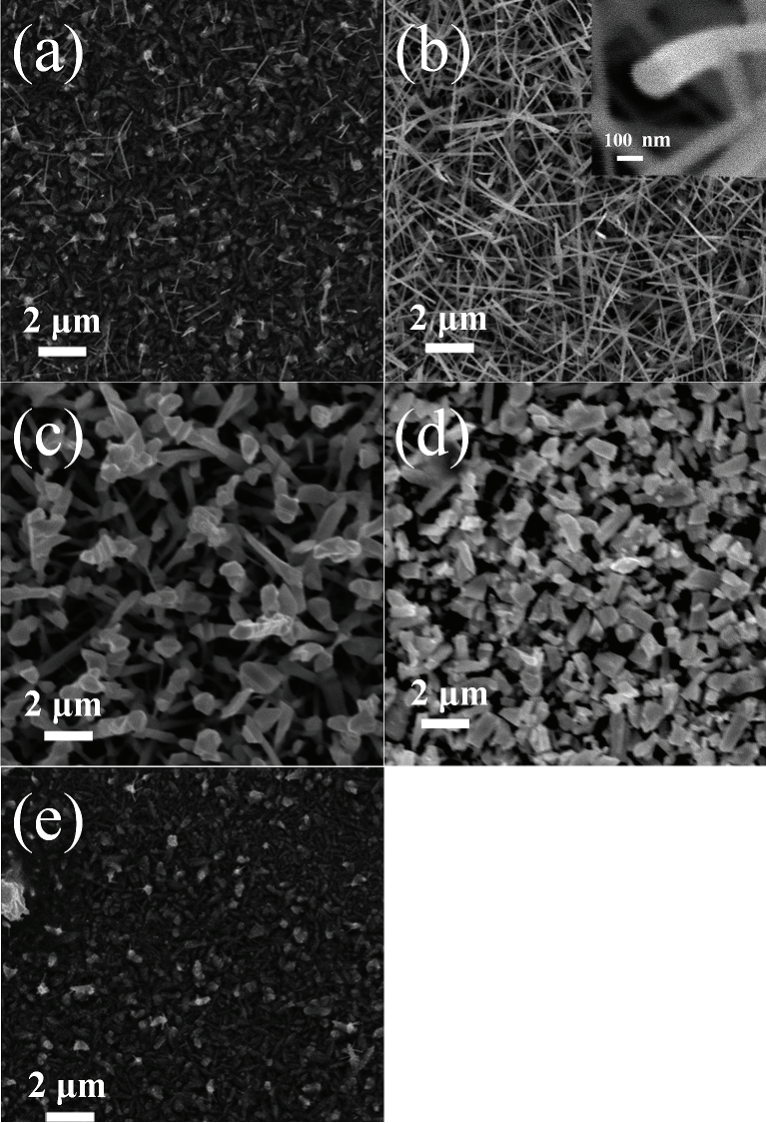
**SEM images of the tungsten oxide nanostructures**. SEM images of the tungsten oxide nanostructures grown in the different substrate temperature zones.

Figure 2a,b,c,d,e shows SEM images of the tungsten oxide nanostructures synthesized at temperature zones 1 to 5 (Figure 1), respectively. A pad tungsten oxide layer and a very low density of tungsten oxide whiskers oriented in random directions on the pad tungsten oxide layer in zone 1 were observed (Figure 2a), which suggests that the two-dimensional (2D) nanostructures formed first on the Si substrate and subsequently 1D nanostructures formed on the pregrown 2D nanostructures. The diameters and lengths of the whiskers were in the range of a few tens of nanometers and 0.5 to 2 μm, respectively. High-density fine needle-like tungsten oxide nanowires oriented in random directions were observed in zone 2 (Figure 2b). The diameters and lengths of these nanowires were in the range of a few tens to a few hundreds of nanometers and 5 to 10 μm, respectively. The nanowires were oriented randomly, and some appeared to be connected to each other. Larva-like nanostructures were grown in random directions in zone 3 (Figure 2c). They were partially networked by the growth of secondary dendrites. The nanostructures were not uniform in diameter. The diameters of the nanostructures ranged from 0.2 to 1.5 μm, and the lengths were in the range of 3 to 6 μm. Each nanostructure had several nodes like a larva. The nanostructures grown in zone 4 had a very short rod-like morphology with a rectangular or square cross-section (Figure 2d). They were particles with an orthorhombic shape; the edge lengths of which were in the range of 1 to 3 μm. A tungsten oxide film thicker than the pad tungsten oxide grown in zone 1 was grown again in zone 5 (Figure 2e). Based on the SEM images of the nanostructures grown in the different temperature zones, the individual nanostructures appear to change from a longer, thinner needle-like wire morphology to a shorter, thicker rod-like morphology as the substrate temperature was increased.

**Figure 3:**
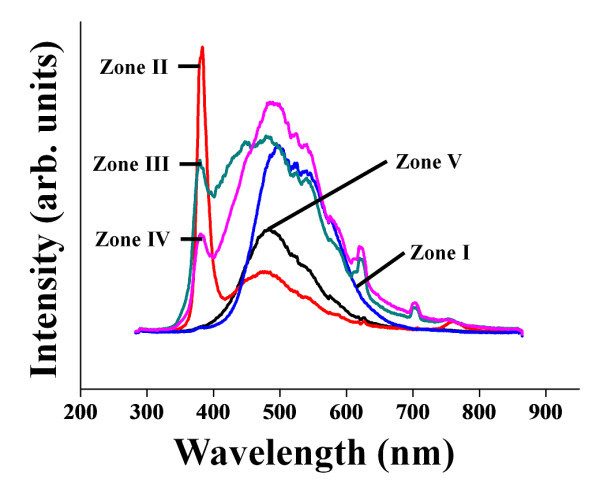
**PL spectra of the nanostructures**. PL spectra of the tungsten oxide nanostructures grown in the different substrate temperature zones.

**Figure 4:**
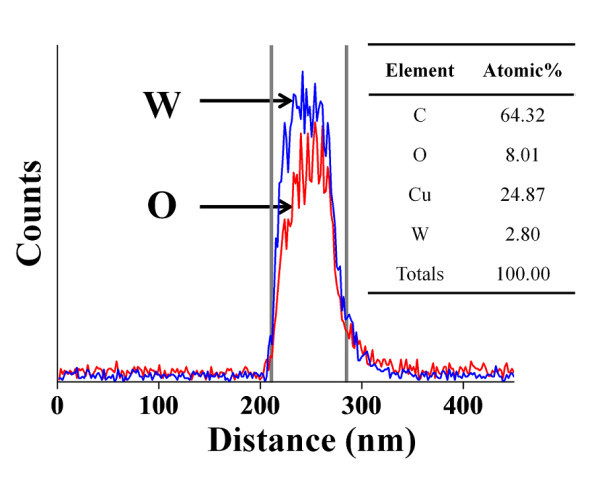
**EDS line scanning profile**. TEM-EDX line concentration profiles of W and O along the line drawn across the diameter of a typical tungsten oxide nanowire synthesized by a catalyst-free thermal evaporation method. Cu and C in the inset table are due to TEM grid.

Figure 3 shows the PL spectra of the nanostructures synthesized at five different substrate temperature zones. A relatively strong broad blue emission band centered at approximately 475 nm, and several shoulders exist in the spectrum of the nanostructures synthesized in zone 1. This blue emission might be attributed to the band-to-band emission, as suggested by Niederberger *et al*. [7] and Luo *et al*. [8], because the photon energy 2.61 eV corresponding to the wavelength of the blue emission falls in the range of the indirect energy gap of tungsten oxide corresponding to 475 nm. This is in good agreement with previous reports. Chang *et al*. [11] observed a strong blue emission peak at approximately 470 nm in the PL spectrum of nitrogen-doped tungsten oxide nanowires synthesized by reducing the tungsten oxide source with NH_3 _gas on a Si wafer. Luo *et al*. [8] also reported strong blue emission band centered at 467 nm from tungsten oxide nanowire networks. In contrast, a sharp strong NUV emission band at 390 nm and a broad weak blue emission band centered approximately at 475 nm from the needle-like nanostructures grown in zone 2 were observed in this study. The strong NUV emission from our tungsten oxide nanowires synthesized in zone 2 can be explained by a combination of the following two sources:

1. Oxygen vacancies: The NUV emission is attributed to the localized states of oxygen vacancies in the conduction band of the needle-like tungsten oxide nanostructures. Luo *et al*. [8] reported an NUV emission band centered at 395 nm from WO_3 - *x *_nanowire networks, even if the emission band was not as sharp and strong as the one from the needle-like tungsten oxide nanostructures synthesized in this work. They attributed the NUV emission to the states of oxygen vacancies in the conduction band of WO_3 - *x *_nanowire networks. They also demonstrated using SEM and X-ray photoemission spectroscopy analyses that oxygen vacancies existed in the WO_3 - *x *_nanowire network but not in the WO_3 _nanowire network. Needle-like tungsten oxide nanostructures were grown in zone 2 (590°C to 750°C), i.e., in quite a low-temperature range. The W/O atomic ratio (8.01/2.80) in the needle-like tungsten nanostructures is approximately 2.86 as shown in the energy-dispersive X-ray spectroscopy (EDS) line scanning profile (Figure 4), so that the nanostructures do not have a molecular formula of WO_3 _but of WO_3 - *x*_. This may be due to the relatively low process temperature for the tungsten nanowire synthesis. The tungsten oxide nanostructures grown at low temperatures have been reported to commonly possess more defects such as oxygen vacancies [9]. Therefore, the NUV emission from the needle-like tungsten nanostructures was attributed to the localized states of oxygen vacancies, as Luo *et al*. [8] suggested.

2. Surface states: The needle-like nanostructures obviously have a far higher surface state density than other nanostructures, such as thicker nanorods and thin films synthesized in zones 1, 3, 4, and 5. Therefore, the far stronger NUV emission from the needle-like nanostructures than that from the WO_3 - *x *_nanowire networks in Luo *et al*'s report [8] may be due partially to the higher density of surface states at the surfaces of the needle-like nanostructures.

The PL spectra showed that the NUV emission intensity tends to decrease with increasing substrate temperature, but the blue emission intensity tends to increase. This tendency appears to depend on the morphology of the tungsten oxide nanostructures because the morphology of the nanostructures also changes from whiskers to nanoneedles and nanorods to thin films. In other words, the surface-to-volume ratio of the nanostructures decreases with increasing substrate temperature. In addition, the oxide nanostructures synthesized at low temperatures commonly possess more oxygen vacancies. Therefore, the blue luminescence is predominant in tungsten oxide nanostructures with a low oxygen vacancy concentration and low surface-to-volume ratios synthesized at higher temperatures. This suggests that the blue emission does not originate from deep level defects but from a band-to-band transition. The strong blue emission obtained in the lowest temperature zone (zone 1) is presumably due to the low surface-to-volume ratio of the pad tungsten oxide layer with a thin film morphology synthesized in such a low-temperature range.

## Conclusions

In summary, intense NUV emission was obtained from the needle-like WO_3 _nanostructures synthesized in the temperature range of 590°C to 750°C by the thermal evaporation of WO_3 _powders. The NUV emission might be due to localized states associated with oxygen vacancies and surface states.

## Competing interests

The authors declare that they have no competing interests.

## Authors' contributions

SP carried out the SEM and XRD analyses. HK SP carried out the TEM analysis. CJ performed the PL analysis. CL conceived of the study, and participated in its design, coordination, and drafting the manuscript. All authors read and approved the final manuscript.
